# Dicarbon­yl(η^5^-cyclo­penta­dien­yl)bis­(trimethyl­phosphine)molybdenum(II) trifluoro­methane­sulfonate

**DOI:** 10.1107/S1600536808032662

**Published:** 2008-10-15

**Authors:** Samuel Jali, Holger B. Friedrich, Muhammad D. Bala

**Affiliations:** a School of Chemistry, University of KwaZulu-Natal, Westville Campus, Private Bag X54001, Durban 4000, South Africa

## Abstract

In the title compound, [Mo(C_5_H_5_)(CO)_2_(C_3_H_9_P)_2_]CF_3_SO_3_, the cationic complex displays a classical four-legged piano-stool square-pyramidal geometry with a *trans* configuration of the basal ligands around the Mo atom. The cyclo­penta­dienyl (Cp) ligand occupies the apical position of the piano-stool configuration. The average Mo—P bond length of the two *trans* PMe_3_ ligands is 2.474 (5) Å and the Mo—Cp centroid distance is 2.003 (2) Å.

## Related literature

For similar crystal structures, see: Fettinger *et al.* (1998 ▶); Schubert *et al.* (1982 ▶). For general discussion of piano-stool complexes, see: Kubáček *et al.* (1982 ▶); Haines *et al.* (1967 ▶); Treichel *et al.* (1967 ▶). For our previous work in this area, see: Changamu *et al.* (2006 ▶). For the synthesis of the starting compound, see: Markham *et al.* (1985 ▶). 
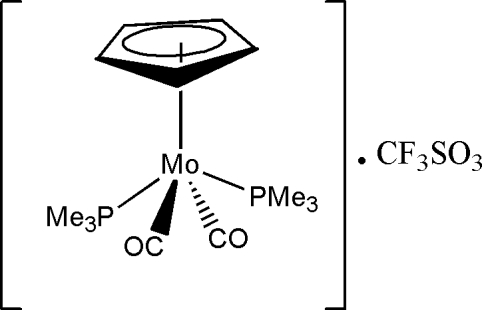


## Experimental

### 

#### Crystal data

[Mo(C_5_H_5_)(CO)_2_(C_3_H_9_P)_2_]CF_3_SO_3_
*M* *_r_* = 518.26
Monoclinic, 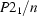

*a* = 10.8919 (5) Å
*b* = 8.0864 (3) Å
*c* = 23.4132 (10) Å
β = 95.464 (1)°
*V* = 2052.78 (15) Å^3^
*Z* = 4
Mo *K*α radiation
μ = 0.94 mm^−1^
*T* = 296 (2) K
0.48 × 0.23 × 0.16 mm

#### Data collection

Bruker SMART CCD area-detector diffractometer
Absorption correction: integration (*XPREP*; Bruker, 2005 ▶) *T*_min_ = 0.660, *T*_max_ = 0.864
20835 measured reflections
4960 independent reflections
4542 reflections with *I* > 2σ(*I*)
*R*_int_ = 0.042

#### Refinement

*R*[*F* ^2^ > 2σ(*F* ^2^)] = 0.024
*wR*(*F* ^2^) = 0.054
*S* = 1.09
4960 reflections
241 parameters
H-atom parameters constrained
Δρ_max_ = 0.43 e Å^−3^
Δρ_min_ = −0.48 e Å^−3^

### 

Data collection: *APEX2* (Bruker, 2005 ▶); cell refinement: *SAINT-Plus* (Bruker, 2005 ▶); data reduction: *SAINT-Plus*; program(s) used to solve structure: *SHELXTL* (Sheldrick, 2008 ▶); program(s) used to refine structure: *SHELXTL*; molecular graphics: *PLATON* (Spek, 2003 ▶) and *ORTEP-3* (Farrugia, 1997 ▶); software used to prepare material for publication: *SHELXTL*.

## Supplementary Material

Crystal structure: contains datablocks global, I. DOI: 10.1107/S1600536808032662/dn2388sup1.cif

Structure factors: contains datablocks I. DOI: 10.1107/S1600536808032662/dn2388Isup2.hkl

Additional supplementary materials:  crystallographic information; 3D view; checkCIF report

## Figures and Tables

**Fig. 1.**
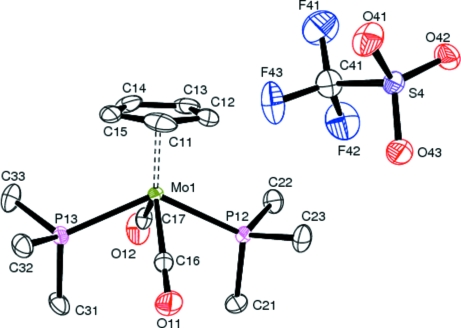
Molecular structure of the title complex with the atom labelling scheme. Hydrogen atoms are omitted for clarity. Ellipsoids are drawn at the 50% probability level.

## supplementary crystallographic information

### Comment

The title compound was prepared as part of our ongoing study on mixed ligand complexes (Changamu *et al.*, 2006). It crystallizes in a monoclinic *P*2_1_/*n* space group having a square-pyramidal geometry of ligands around Mo typical of four-legged piano-stool CpMo*L*_4_ complexes (Kubáček *et al.*, 1982; Fettinger *et al.*,1998). The complex shows a *trans* orientation of the two phosphine ligands.

A cation-anion paired complex [Mo(C_5_H_5_)(CO)_2_(PMe_3_)_2_][Mo(C_5_H_5_)(CO)_3_] also having a similar arrangement of ligands around the cationic moiety has been previously reported (Schubert *et al.*, 1982).

The title compound forms an adduct pair with trifluorosulphonic acid (trifluoromethanesulfonate) group. Generally these complexes may be considered as disubstituted derivatives of [CpMo(CO)_4_]^+^ in which double CO-substitution leads to either a *cis* or a *trans* product (Treichel *et al.*,1967; Haines *et al.*, 1967). However, due to unfavourable steric interactions between bulky ligands only the *trans* isomer was observed in the title complex. The influence of steric size on the spatial orientation of the ligands is further evidenced by the large difference in the angles between the dissimilar *trans* ligands. Hence the P12—Mo1—P13 bonds angle is 130.34 (4)° while the C16—Mo1—C17 bonds angle is only 110.75 (4)°. The crystal of (I) is stabilized by a series of short inter-molecular contacts between neighbouring molecules.

### Experimental

To a dark purple solution of 0.85 g (2.15 mmol) of [CpMo(CO)_3_][SO_3_CF_3_] (Markham *et al.*, 1985) in dichloromethane was added 1 ml (0.735 g, 9.66 mmol) of PMe_3_ at room temperature. The reaction was stirred overnight. The resulting yellow solution was transferred to an ether–hexane mixture (10: 1), cooled to -78°C. A yellow solid precipitate resulted. Room temperature recrystallization from hexane–dichloromethane afforded yellow crystals of (I) suitable for X-ray analysis.

### Refinement

All H atoms attached to C atoms were fixed geometrically and treated as riding with C—H = 0.98 Å (methyl) and 0.95 Å (Cp) with U_iso_(H) = 1.2U_eq_(Cp) or U_iso_(H) = 1.5U_eq_(methyl).

### Crystal data

**Table:**

[Mo(C_5_H_5_)(CO)_2_(C_3_H_9_P)_2_]CF_3_SO_3_	*F*(000) = 1048
*M**_r_* = 518.26	*D*_x_ = 1.677 Mg m^−^^3^
Monoclinic, *P*2_1_/*n*	Mo *K*α radiation, λ = 0.71073 Å
Hall symbol: -P 2yn	Cell parameters from 6234 reflections
*a* = 10.8919 (5) Å	θ = 1.8–28.0°
*b* = 8.0864 (3) Å	µ = 0.94 mm^−^^1^
*c* = 23.4132 (10) Å	*T* = 296 K
β = 95.464 (1)°	Block, yellow
*V* = 2052.78 (15) Å^3^	0.48 × 0.23 × 0.16 mm
*Z* = 4	

### Data collection

**Table:**

Bruker SMART CCD area-detector diffractometer	4960 independent reflections
Radiation source: fine-focus sealed tube	4542 reflections with *I* > 2σ(*I*)
graphite	*R*_int_ = 0.042
φ and ω scans	θ_max_ = 28.0°, θ_min_ = 1.8°
Absorption correction: integration (SADABS; Bruker, 2005)	*h* = −14→14
*T*_min_ = 0.660, *T*_max_ = 0.864	*k* = −10→10
20835 measured reflections	*l* = −29→30

### Refinement

**Table:**

Refinement on *F*^2^	Primary atom site location: structure-invariant direct methods
Least-squares matrix: full	Secondary atom site location: difference Fourier map
*R*[*F*^2^ > 2σ(*F*^2^)] = 0.024	Hydrogen site location: inferred from neighbouring sites
*wR*(*F*^2^) = 0.054	H-atom parameters constrained
*S* = 1.09	*w* = 1/[σ^2^(*F*_o_^2^) + (0.0157*P*)^2^ + 1.38*P*] where *P* = (*F*_o_^2^ + 2*F*_c_^2^)/3
4960 reflections	(Δ/σ)_max_ = 0.003
241 parameters	Δρ_max_ = 0.43 e Å^−^^3^
0 restraints	Δρ_min_ = −0.48 e Å^−^^3^

### Special details

**Table:**

Geometry. All e.s.d.'s (except the e.s.d. in the dihedral angle between two l.s. planes) are estimated using the full covariance matrix. The cell e.s.d.'s are taken into account individually in the estimation of e.s.d.'s in distances, angles and torsion angles; correlations between e.s.d.'s in cell parameters are only used when they are defined by crystal symmetry. An approximate (isotropic) treatment of cell e.s.d.'s is used for estimating e.s.d.'s involving l.s. planes.
Refinement. Refinement of *F*^2^ against ALL reflections. The weighted *R*-factor *wR* and goodness of fit *S* are based on *F*^2^, conventional *R*-factors *R* are based on *F*, with *F* set to zero for negative *F*^2^. The threshold expression of *F*^2^ > σ(*F*^2^) is used only for calculating *R*-factors(gt) *etc*. and is not relevant to the choice of reflections for refinement. *R*-factors based on *F*^2^ are statistically about twice as large as those based on *F*, and *R*- factors based on ALL data will be even larger.

### Fractional atomic coordinates and isotropic or equivalent isotropic displacement parameters (Å^2^)

**Table:**

	*x*	*y*	*z*	*U*_iso_*/*U*_eq_	
Mo1	0.085336 (12)	0.084776 (17)	0.865019 (6)	0.01416 (4)	
S4	0.41202 (4)	0.53406 (6)	1.12198 (2)	0.02194 (10)	
P12	0.29392 (4)	−0.03890 (6)	0.87511 (2)	0.01945 (10)	
P13	−0.11979 (4)	−0.04769 (6)	0.84825 (2)	0.01899 (10)	
O11	0.07705 (14)	−0.1616 (2)	0.96710 (6)	0.0382 (4)	
O12	0.10322 (15)	−0.1046 (2)	0.75064 (7)	0.0429 (4)	
O41	0.33084 (14)	0.6492 (2)	1.14629 (7)	0.0394 (4)	
O42	0.53050 (12)	0.59896 (17)	1.11112 (7)	0.0318 (3)	
O43	0.41363 (13)	0.37125 (19)	1.14704 (7)	0.0341 (3)	
F41	0.33337 (16)	0.6369 (2)	1.01893 (6)	0.0600 (4)	
F42	0.39519 (15)	0.3859 (2)	1.02214 (6)	0.0547 (4)	
F43	0.22046 (12)	0.4479 (2)	1.05128 (6)	0.0522 (4)	
C11	0.0608 (2)	0.3282 (2)	0.91851 (10)	0.0372 (5)	
H11	0.0451	0.3259	0.9577	0.045*	
C12	0.17744 (19)	0.3326 (2)	0.89736 (10)	0.0322 (5)	
H12	0.2547	0.3324	0.9199	0.039*	
C13	0.1599 (2)	0.3372 (2)	0.83737 (10)	0.0350 (5)	
H13	0.2233	0.3416	0.8122	0.042*	
C14	0.0330 (2)	0.3343 (3)	0.82086 (11)	0.0382 (5)	
H14	−0.0049	0.3363	0.7826	0.046*	
C15	−0.02827 (19)	0.3280 (2)	0.87089 (12)	0.0388 (6)	
H15	−0.1151	0.3242	0.8723	0.047*	
C16	0.08055 (16)	−0.0733 (2)	0.92869 (8)	0.0222 (4)	
C17	0.09558 (16)	−0.0397 (2)	0.79389 (8)	0.0236 (4)	
C21	0.2917 (2)	−0.2612 (2)	0.86869 (12)	0.0390 (5)	
H21A	0.3764	−0.3033	0.8727	0.059*	
H21B	0.2514	−0.2924	0.8310	0.059*	
H21C	0.2460	−0.3085	0.8989	0.059*	
C22	0.39704 (18)	0.0273 (3)	0.82312 (9)	0.0312 (4)	
H22A	0.4707	−0.0430	0.8263	0.047*	
H22B	0.4213	0.1425	0.8306	0.047*	
H22C	0.3549	0.0181	0.7844	0.047*	
C23	0.38690 (19)	−0.0031 (3)	0.94249 (9)	0.0359 (5)	
H23A	0.3408	−0.0378	0.9744	0.054*	
H23B	0.4067	0.1148	0.9463	0.054*	
H23C	0.4634	−0.0671	0.9432	0.054*	
C31	−0.11193 (18)	−0.2684 (2)	0.83587 (10)	0.0320 (5)	
H31A	−0.1953	−0.3151	0.8329	0.048*	
H31B	−0.0619	−0.3207	0.8679	0.048*	
H31C	−0.0743	−0.2890	0.8001	0.048*	
C32	−0.21945 (17)	−0.0306 (3)	0.90569 (9)	0.0274 (4)	
H32A	−0.2405	0.0858	0.9111	0.041*	
H32B	−0.1767	−0.0742	0.9413	0.041*	
H32C	−0.2951	−0.0942	0.8958	0.041*	
C33	−0.21636 (19)	0.0275 (3)	0.78623 (9)	0.0340 (5)	
H33A	−0.2929	−0.0368	0.7816	0.051*	
H33B	−0.1724	0.0154	0.7518	0.051*	
H33C	−0.2358	0.1443	0.7918	0.051*	
C41	0.3370 (2)	0.4999 (3)	1.04982 (9)	0.0338 (5)	

### Atomic displacement parameters (Å^2^)

**Table:**

	*U*^11^	*U*^22^	*U*^33^	*U*^12^	*U*^13^	*U*^23^
Mo1	0.01406 (7)	0.01252 (7)	0.01594 (7)	0.00108 (5)	0.00169 (5)	0.00056 (5)
S4	0.01749 (19)	0.0219 (2)	0.0267 (2)	0.00263 (16)	0.00369 (17)	−0.00058 (17)
P12	0.0156 (2)	0.0171 (2)	0.0252 (2)	0.00222 (16)	−0.00038 (17)	0.00011 (17)
P13	0.0158 (2)	0.0189 (2)	0.0222 (2)	−0.00024 (16)	0.00109 (17)	−0.00368 (17)
O43	0.0266 (7)	0.0312 (8)	0.0444 (9)	−0.0025 (6)	0.0025 (6)	0.0111 (7)
O42	0.0240 (7)	0.0291 (7)	0.0429 (8)	−0.0053 (6)	0.0064 (6)	0.0003 (6)
O11	0.0392 (8)	0.0433 (9)	0.0321 (8)	−0.0005 (7)	0.0036 (7)	0.0186 (7)
O41	0.0342 (8)	0.0435 (9)	0.0409 (9)	0.0151 (7)	0.0064 (7)	−0.0108 (7)
O12	0.0372 (8)	0.0633 (11)	0.0285 (8)	0.0020 (8)	0.0053 (7)	−0.0207 (8)
F42	0.0582 (9)	0.0639 (10)	0.0435 (8)	0.0006 (8)	0.0121 (7)	−0.0247 (7)
F43	0.0294 (7)	0.0817 (11)	0.0439 (8)	−0.0119 (7)	−0.0053 (6)	−0.0059 (8)
F41	0.0748 (11)	0.0613 (10)	0.0415 (8)	−0.0009 (9)	−0.0075 (8)	0.0212 (8)
C14	0.0466 (13)	0.0209 (10)	0.0458 (13)	0.0084 (9)	−0.0025 (11)	0.0122 (9)
C12	0.0299 (10)	0.0149 (9)	0.0508 (13)	−0.0020 (8)	−0.0020 (9)	−0.0052 (9)
C21	0.0284 (10)	0.0194 (10)	0.0687 (16)	0.0076 (8)	0.0017 (10)	−0.0008 (10)
C31	0.0248 (9)	0.0219 (9)	0.0497 (13)	−0.0063 (8)	0.0061 (9)	−0.0119 (9)
C11	0.0564 (14)	0.0157 (9)	0.0429 (12)	−0.0026 (9)	0.0228 (11)	−0.0097 (8)
C41	0.0334 (11)	0.0375 (12)	0.0305 (11)	−0.0011 (9)	0.0034 (9)	−0.0002 (9)
C15	0.0217 (9)	0.0141 (9)	0.0820 (18)	0.0046 (7)	0.0123 (10)	−0.0014 (10)
C17	0.0167 (8)	0.0299 (10)	0.0241 (9)	0.0026 (7)	0.0021 (7)	−0.0020 (8)
C32	0.0225 (9)	0.0294 (10)	0.0315 (10)	−0.0022 (8)	0.0091 (8)	−0.0018 (8)
C16	0.0192 (8)	0.0237 (9)	0.0235 (9)	−0.0004 (7)	0.0009 (7)	0.0008 (7)
C22	0.0224 (9)	0.0383 (11)	0.0339 (11)	0.0003 (8)	0.0089 (8)	−0.0013 (9)
C33	0.0246 (9)	0.0478 (13)	0.0279 (10)	0.0080 (9)	−0.0055 (8)	−0.0055 (9)
C13	0.0372 (11)	0.0162 (9)	0.0549 (14)	0.0009 (8)	0.0217 (10)	0.0089 (9)
C23	0.0265 (10)	0.0458 (13)	0.0331 (11)	0.0017 (9)	−0.0088 (9)	0.0016 (10)

### Geometric parameters (Å, °)

**Table:**

Mo1—C17	1.9581 (19)	C14—H14	0.9500
Mo1—C16	1.9683 (19)	C12—C13	1.400 (3)
Mo1—C13	2.3123 (19)	C12—C11	1.407 (3)
Mo1—C14	2.314 (2)	C12—H12	0.9500
Mo1—C15	2.3346 (19)	C21—H21A	0.9800
Mo1—C12	2.3348 (19)	C21—H21B	0.9800
Mo1—C11	2.362 (2)	C21—H21C	0.9800
Mo1—P12	2.4729 (5)	C31—H31A	0.9800
Mo1—P13	2.4751 (5)	C31—H31B	0.9800
S4—O42	1.4379 (14)	C31—H31C	0.9800
S4—O41	1.4384 (15)	C11—C15	1.406 (3)
S4—O43	1.4408 (15)	C11—H11	0.9500
S4—C41	1.826 (2)	C15—H15	0.9500
P12—C21	1.804 (2)	C32—H32A	0.9800
P12—C22	1.814 (2)	C32—H32B	0.9800
P12—C23	1.815 (2)	C32—H32C	0.9800
P13—C31	1.812 (2)	C22—H22A	0.9800
P13—C32	1.8122 (19)	C22—H22B	0.9800
P13—C33	1.815 (2)	C22—H22C	0.9800
O11—C16	1.152 (2)	C33—H33A	0.9800
O12—C17	1.150 (2)	C33—H33B	0.9800
F42—C41	1.323 (3)	C33—H33C	0.9800
F43—C41	1.341 (3)	C13—H13	0.9500
F41—C41	1.321 (3)	C23—H23A	0.9800
C14—C13	1.399 (3)	C23—H23B	0.9800
C14—C15	1.403 (3)	C23—H23C	0.9800
			
C17—Mo1—C16	108.55 (8)	C11—C12—H12	125.9
C17—Mo1—C13	99.54 (8)	Mo1—C12—H12	120.6
C16—Mo1—C13	145.16 (8)	P12—C21—H21A	109.5
C17—Mo1—C14	95.73 (8)	P12—C21—H21B	109.5
C16—Mo1—C14	151.85 (8)	H21A—C21—H21B	109.5
C13—Mo1—C14	35.22 (8)	P12—C21—H21C	109.5
C17—Mo1—C15	123.82 (9)	H21A—C21—H21C	109.5
C16—Mo1—C15	116.75 (8)	H21B—C21—H21C	109.5
C13—Mo1—C15	58.33 (7)	P13—C31—H31A	109.5
C14—Mo1—C15	35.14 (9)	P13—C31—H31B	109.5
C17—Mo1—C12	131.26 (8)	H31A—C31—H31B	109.5
C16—Mo1—C12	110.69 (8)	P13—C31—H31C	109.5
C13—Mo1—C12	35.06 (8)	H31A—C31—H31C	109.5
C14—Mo1—C12	58.41 (8)	H31B—C31—H31C	109.5
C15—Mo1—C12	58.10 (7)	C15—C11—C12	107.4 (2)
C17—Mo1—C11	153.87 (8)	C15—C11—Mo1	71.51 (11)
C16—Mo1—C11	97.31 (8)	C12—C11—Mo1	71.51 (11)
C13—Mo1—C11	58.21 (8)	C15—C11—H11	126.3
C14—Mo1—C11	58.30 (9)	C12—C11—H11	126.3
C15—Mo1—C11	34.84 (9)	Mo1—C11—H11	122.4
C12—Mo1—C11	34.86 (7)	F41—C41—F42	107.85 (19)
C17—Mo1—P12	75.35 (5)	F41—C41—F43	107.34 (19)
C16—Mo1—P12	75.84 (5)	F42—C41—F43	107.02 (19)
C13—Mo1—P12	92.28 (5)	F41—C41—S4	111.54 (16)
C14—Mo1—P12	125.47 (6)	F42—C41—S4	111.43 (15)
C15—Mo1—P12	145.32 (5)	F43—C41—S4	111.42 (15)
C12—Mo1—P12	87.35 (5)	C14—C15—C11	108.34 (19)
C11—Mo1—P12	115.83 (6)	C14—C15—Mo1	71.62 (11)
C17—Mo1—P13	76.49 (5)	C11—C15—Mo1	73.65 (11)
C16—Mo1—P13	75.55 (5)	C14—C15—H15	125.8
C13—Mo1—P13	132.34 (6)	C11—C15—H15	125.8
C14—Mo1—P13	97.26 (6)	Mo1—C15—H15	120.6
C15—Mo1—P13	84.17 (5)	O12—C17—Mo1	176.13 (18)
C12—Mo1—P13	140.93 (5)	P13—C32—H32A	109.5
C11—Mo1—P13	107.38 (6)	P13—C32—H32B	109.5
P12—Mo1—P13	130.342 (16)	H32A—C32—H32B	109.5
O42—S4—O41	115.43 (10)	P13—C32—H32C	109.5
O42—S4—O43	115.41 (9)	H32A—C32—H32C	109.5
O41—S4—O43	114.49 (10)	H32B—C32—H32C	109.5
O42—S4—C41	102.81 (10)	O11—C16—Mo1	177.79 (17)
O41—S4—C41	103.07 (10)	P12—C22—H22A	109.5
O43—S4—C41	103.04 (10)	P12—C22—H22B	109.5
C21—P12—C22	104.03 (11)	H22A—C22—H22B	109.5
C21—P12—C23	103.48 (11)	P12—C22—H22C	109.5
C22—P12—C23	101.91 (10)	H22A—C22—H22C	109.5
C21—P12—Mo1	112.96 (7)	H22B—C22—H22C	109.5
C22—P12—Mo1	116.01 (7)	P13—C33—H33A	109.5
C23—P12—Mo1	116.75 (8)	P13—C33—H33B	109.5
C31—P13—C32	103.58 (10)	H33A—C33—H33B	109.5
C31—P13—C33	103.56 (11)	P13—C33—H33C	109.5
C32—P13—C33	103.05 (10)	H33A—C33—H33C	109.5
C31—P13—Mo1	113.33 (7)	H33B—C33—H33C	109.5
C32—P13—Mo1	116.40 (7)	C14—C13—C12	108.3 (2)
C33—P13—Mo1	115.32 (8)	C14—C13—Mo1	72.45 (12)
C13—C14—C15	107.8 (2)	C12—C13—Mo1	73.35 (11)
C13—C14—Mo1	72.33 (11)	C14—C13—H13	125.9
C15—C14—Mo1	73.24 (12)	C12—C13—H13	125.9
C13—C14—H14	126.1	Mo1—C13—H13	120.1
C15—C14—H14	126.1	P12—C23—H23A	109.5
Mo1—C14—H14	120.2	P12—C23—H23B	109.5
C13—C12—C11	108.2 (2)	H23A—C23—H23B	109.5
C13—C12—Mo1	71.59 (11)	P12—C23—H23C	109.5
C11—C12—Mo1	73.63 (11)	H23A—C23—H23C	109.5
C13—C12—H12	125.9	H23B—C23—H23C	109.5
			
C17—Mo1—P12—C21	−57.17 (11)	C13—Mo1—C12—C11	116.16 (19)
C16—Mo1—P12—C21	56.73 (11)	C14—Mo1—C12—C11	78.67 (15)
C13—Mo1—P12—C21	−156.44 (11)	C15—Mo1—C12—C11	37.08 (14)
C14—Mo1—P12—C21	−143.76 (12)	P12—Mo1—C12—C11	−146.07 (14)
C15—Mo1—P12—C21	173.54 (15)	P13—Mo1—C12—C11	20.12 (18)
C12—Mo1—P12—C21	168.84 (11)	C13—C12—C11—C15	0.8 (2)
C11—Mo1—P12—C21	148.08 (11)	Mo1—C12—C11—C15	−62.93 (14)
P13—Mo1—P12—C21	0.23 (10)	C13—C12—C11—Mo1	63.70 (14)
C17—Mo1—P12—C22	62.81 (10)	C17—Mo1—C11—C15	44.2 (2)
C16—Mo1—P12—C22	176.71 (10)	C16—Mo1—C11—C15	−127.59 (13)
C13—Mo1—P12—C22	−36.45 (10)	C13—Mo1—C11—C15	79.01 (14)
C14—Mo1—P12—C22	−23.77 (11)	C14—Mo1—C11—C15	37.34 (13)
C15—Mo1—P12—C22	−66.48 (14)	C12—Mo1—C11—C15	116.36 (19)
C12—Mo1—P12—C22	−71.18 (10)	P12—Mo1—C11—C15	154.64 (11)
C11—Mo1—P12—C22	−91.94 (10)	P13—Mo1—C11—C15	−50.51 (13)
P13—Mo1—P12—C22	120.21 (8)	C17—Mo1—C11—C12	−72.2 (2)
C17—Mo1—P12—C23	−176.98 (11)	C16—Mo1—C11—C12	116.05 (14)
C16—Mo1—P12—C23	−63.08 (11)	C13—Mo1—C11—C12	−37.35 (13)
C13—Mo1—P12—C23	83.76 (11)	C14—Mo1—C11—C12	−79.02 (15)
C14—Mo1—P12—C23	96.44 (12)	C15—Mo1—C11—C12	−116.36 (19)
C15—Mo1—P12—C23	53.74 (15)	P12—Mo1—C11—C12	38.28 (15)
C12—Mo1—P12—C23	49.03 (11)	P13—Mo1—C11—C12	−166.87 (12)
C11—Mo1—P12—C23	28.27 (11)	O42—S4—C41—F41	−56.27 (18)
P13—Mo1—P12—C23	−119.58 (9)	O41—S4—C41—F41	64.05 (18)
C17—Mo1—P13—C31	51.37 (10)	O43—S4—C41—F41	−176.57 (16)
C16—Mo1—P13—C31	−62.19 (10)	O42—S4—C41—F42	64.33 (18)
C13—Mo1—P13—C31	142.04 (11)	O41—S4—C41—F42	−175.35 (16)
C14—Mo1—P13—C31	145.55 (11)	O43—S4—C41—F42	−55.97 (18)
C15—Mo1—P13—C31	178.23 (11)	O42—S4—C41—F43	−176.21 (16)
C12—Mo1—P13—C31	−167.35 (12)	O41—S4—C41—F43	−55.89 (18)
C11—Mo1—P13—C31	−155.46 (10)	O43—S4—C41—F43	63.49 (18)
P12—Mo1—P13—C31	−5.58 (9)	C13—C14—C15—C11	0.4 (2)
C17—Mo1—P13—C32	171.34 (10)	Mo1—C14—C15—C11	65.00 (14)
C16—Mo1—P13—C32	57.78 (10)	C13—C14—C15—Mo1	−64.55 (14)
C13—Mo1—P13—C32	−97.99 (11)	C12—C11—C15—C14	−0.8 (2)
C14—Mo1—P13—C32	−94.48 (10)	Mo1—C11—C15—C14	−63.68 (14)
C15—Mo1—P13—C32	−61.79 (10)	C12—C11—C15—Mo1	62.93 (14)
C12—Mo1—P13—C32	−47.38 (12)	C17—Mo1—C15—C14	−42.02 (16)
C11—Mo1—P13—C32	−35.49 (10)	C16—Mo1—C15—C14	177.95 (13)
P12—Mo1—P13—C32	114.39 (8)	C13—Mo1—C15—C14	37.68 (14)
C17—Mo1—P13—C33	−67.75 (10)	C12—Mo1—C15—C14	79.19 (15)
C16—Mo1—P13—C33	178.69 (10)	C11—Mo1—C15—C14	116.29 (19)
C13—Mo1—P13—C33	22.92 (11)	P12—Mo1—C15—C14	73.65 (19)
C14—Mo1—P13—C33	26.43 (10)	P13—Mo1—C15—C14	−111.46 (14)
C15—Mo1—P13—C33	59.12 (10)	C17—Mo1—C15—C11	−158.31 (13)
C12—Mo1—P13—C33	73.53 (12)	C16—Mo1—C15—C11	61.66 (14)
C11—Mo1—P13—C33	85.42 (10)	C13—Mo1—C15—C11	−78.61 (14)
P12—Mo1—P13—C33	−124.70 (8)	C14—Mo1—C15—C11	−116.29 (19)
C17—Mo1—C14—C13	−98.44 (15)	C12—Mo1—C15—C11	−37.11 (13)
C16—Mo1—C14—C13	111.66 (19)	P12—Mo1—C15—C11	−42.64 (19)
C15—Mo1—C14—C13	115.5 (2)	P13—Mo1—C15—C11	132.24 (13)
C12—Mo1—C14—C13	37.32 (13)	C15—C14—C13—C12	0.0 (2)
C11—Mo1—C14—C13	78.53 (15)	Mo1—C14—C13—C12	−65.11 (14)
P12—Mo1—C14—C13	−22.36 (17)	C15—C14—C13—Mo1	65.15 (14)
P13—Mo1—C14—C13	−175.50 (13)	C11—C12—C13—C14	−0.5 (2)
C17—Mo1—C14—C15	146.02 (14)	Mo1—C12—C13—C14	64.52 (14)
C16—Mo1—C14—C15	−3.9 (2)	C11—C12—C13—Mo1	−65.03 (14)
C13—Mo1—C14—C15	−115.5 (2)	C17—Mo1—C13—C14	86.41 (15)
C12—Mo1—C14—C15	−78.22 (15)	C16—Mo1—C13—C14	−129.86 (16)
C11—Mo1—C14—C15	−37.02 (13)	C15—Mo1—C13—C14	−37.60 (14)
P12—Mo1—C14—C15	−137.90 (12)	C12—Mo1—C13—C14	−115.95 (19)
P13—Mo1—C14—C15	68.96 (13)	C11—Mo1—C13—C14	−78.82 (15)
C17—Mo1—C12—C13	29.95 (16)	P12—Mo1—C13—C14	161.94 (14)
C16—Mo1—C12—C13	171.56 (12)	P13—Mo1—C13—C14	6.05 (18)
C14—Mo1—C12—C13	−37.49 (13)	C17—Mo1—C13—C12	−157.63 (12)
C15—Mo1—C12—C13	−79.07 (14)	C16—Mo1—C13—C12	−13.9 (2)
C11—Mo1—C12—C13	−116.16 (19)	C14—Mo1—C13—C12	115.95 (19)
P12—Mo1—C12—C13	97.78 (12)	C15—Mo1—C13—C12	78.35 (14)
P13—Mo1—C12—C13	−96.03 (14)	C11—Mo1—C13—C12	37.13 (13)
C17—Mo1—C12—C11	146.11 (14)	P12—Mo1—C13—C12	−82.11 (12)
C16—Mo1—C12—C11	−72.28 (15)	P13—Mo1—C13—C12	122.01 (12)
